# Impacted Fish Bones: A National Survey

**DOI:** 10.7759/cureus.97496

**Published:** 2025-11-22

**Authors:** Jemma Butler, Adan Chew, Neil A Giblett, Andrew Mowat

**Affiliations:** 1 Otolaryngology, Worcestershire Acute Hospitals National Health Service (NHS) Trust, Worcester, GBR; 2 Otolaryngology, Barking, Havering and Redbridge University Hospitals National Health Service (NHS) Trust, London, GBR; 3 Otolaryngology, The Royal Wolverhampton National Health Service (NHS) Trust, Wolverhampton, GBR

**Keywords:** covid 19, fishbone ingestion, flexible fiberoptic nasopharyngolaryngoscopy, lateral neck radiography (lnr), oesophageal foreign body, oesophageal rupture

## Abstract

Background

Impacted fish bones are a common referral to the ear, nose, and throat (ENT) acute on-call service. Diagnosis is often challenging as neither lateral neck radiograph (X-ray) nor flexible nasendoscopy (FNE) is perfectly sensitive. Management dilemmas are common, particularly as bones can result in significant complications.

Objectives

The objective of the study is to assess awareness of current ENT UK guidelines, investigate practice patterns, and identify knowledge gaps in the management of patients presenting with suspected fish bone foreign bodies in the upper aerodigestive tract.

Design

This is a cross-sectional survey study.

Setting

A UK-wide survey was distributed across ENT departments.

Participants

Fifty-three respondents comprise consultants, higher specialty ENT trainees, core surgical trainees, general practice (GP) trainees, and foundation doctors.

Main outcome measures

The main outcome measures are clinician awareness of ENT UK guidelines, confidence in management, preferred investigation pathways, knowledge of radio-opaque versus radiolucent fish species, and common symptoms, findings, and complications associated with fish bone foreign bodies.

Results

Of the 53 clinicians surveyed, 48 (91%) were unaware of any guidelines for fish bone foreign body management, while only three (6%) identified the ENT UK guideline. Confidence in management was not significantly associated with seniority (p = 0.31). Knowledge of radiological characteristics of fish species was variable, with 35 (66%) correctly identifying radio-opaque species and six (11%) exclusively identifying radiolucent species. The most commonly reported presenting symptoms were odynophagia (n = 42, 79%), dysphagia (n = 38, 72%), and unilateral neck pain (n = 31, 58%). The palatine tonsils (n = 29, 55%) and tongue base (n = 24, 45%) were the most frequent anatomical sites for impaction. Computed tomography (CT) imaging was the most commonly selected subsequent investigation when initial assessments (FNE or X-ray) were inconclusive (n = 40, 75%).

Conclusions

This study reveals significant variability in the awareness, investigation, and management of fish bone foreign bodies among ENT clinicians in the UK. There is a need for updated, post-pandemic guidelines alongside targeted education to enhance clinical confidence, reduce unnecessary investigations, and improve patient outcomes.

## Introduction

Fish bone impaction within the upper aerodigestive tract is a frequent referral to the acute ear, nose, and throat (ENT) services, accounting for up to 30% of emergency presentations [1]. Asian populations have higher rates due to fish-based diets, which are often served without deboning. Western populations more commonly present with impacted meat boluses [2]. A male preponderance is reported [2]. An age divide exists in retained fish bone location. Patients under 40 more commonly present with oropharyngeal bones. Older patients (>40 years) are more likely to present with esophageal fish bones, a location associated with a higher risk. Denture use is associated with increased incidence [3], as palatal sensitivity is reduced, leading to swallow incoordination [4]. Hence, there should be a lower threshold to obtain cross-sectional imaging in older populations [4].

Impacted fish bones commonly cause dysphagia, odynophagia, regurgitation, dysphonia, chest pain, back pain, unilateral neck pain, globus, and neck stiffness [2,5-7]. Fish bones frequently lodge in the valleculae, piriform fossae, tongue base, or the palatine tonsils [2]. Fish bones carry the risk of serious complications, including esophageal perforation. The bones are not sterile and may lead to abscess formation and mediastinitis if left [7]. Complication rates as high as 3% have been reported [8].

In cases in which a fish bone cannot be identified on examination, imaging should be conducted comprehensively. Plain radiographs (X-ray) have historically been used as a screening investigation due to their accessibility and low radiation dose. Given the limitations of the history, examination, and lateral neck X-ray, a cohort of patients with persistent foreign body sensation following fish consumption remains. These cases provide a decision-making dilemma, and practice varies. Computed tomography (CT) has consistently shown superior diagnostic accuracy for fish bone foreign bodies compared to lateral neck X-rays [5,9-12].

Once an impacted fish bone has been identified, it requires urgent removal. Fish bones lodged in the soft palate or oropharynx are often simple to remove under local anesthesia. Impacted bones around the larynx, or in the upper esophagus, usually require a general anesthetic for retrieval.

During the COVID-19 pandemic, ENT UK released guidelines addressing the diagnosis, risk stratification, and management of fish bone foreign bodies. Attempts were made to reduce the number of flexible nasendoscopies (FNE) performed, to limit aerosol generation [13]. Since the resolution of the pandemic and return to normal working practices, these guidelines have not been updated. ENT UK stratifies fish bone ingestion risk as low (uncertain history, no symptoms), medium (clear history, mild symptoms), and high (airway compromise or severe symptoms), and recommends tailored use of FNE, X-ray, and CT imaging based on the risk level [6].

Despite the clinical frequency of fish bone presentations and the availability of ENT UK guidance, there is little evidence regarding clinician awareness or adherence to these recommendations, and practice appears highly variable. This gap highlights the need to understand current national practice. This study aims to evaluate the awareness of ENT UK guidelines among clinicians, their confidence in managing fish bone foreign bodies, and their understanding of the radio-opacity of various fish species. We developed a questionnaire distributed to ENT clinicians across the United Kingdom, encompassing all levels of training. The authors have observed significant disparities in patient management within their own region and hypothesize that this inconsistency is also prevalent nationally.

## Materials and methods

Study design and setting

A cross-sectional mixed-methods survey was conducted among otolaryngology clinicians across the United Kingdom. This study was reported in accordance with the STROBE (Strengthening the Reporting of Observational Studies in Epidemiology) guidelines. The study aimed to assess awareness, confidence, and practice patterns in the management of suspected fish bone foreign bodies.

Participants and recruitment

All UK-based medical doctors working in ENT, from Foundation Year 1 (F1) to consultant level, including higher specialty trainees, core surgical trainees, and general practice (GP) trainees, were eligible to participate. Doctors not working in ENT were excluded. Recruitment was carried out via mailing lists and social media platforms over a six-week period spanning July and August 2024. Participation was voluntary and anonymous, and the total number of invitations distributed was not recorded.

Data collection

The online questionnaire (Appendix) collected demographic data, including clinical grade, region of practice, and whether respondents had access to a dedicated fish bone management pathway at their institution.

Main outcome measures

The primary outcomes assessed were participants’ awareness of ENT UK guidelines, confidence in investigating and managing suspected cases, preferred investigation and treatment strategies, and their knowledge of the radiographic visibility (radio-opacity) of commonly consumed fish species. Hypothetical clinical scenarios were provided to explore investigation and management preferences across low-, medium-, and high-risk patient groups, based on ENT UK guidelines criteria [7]. Knowledge of fish bone radio-opacity was also assessed by comparing responses with published literature [14,15].

Statistical analysis

Descriptive statistics were used to analyze demographic characteristics, guideline awareness, confidence levels, and clinical decision-making patterns. Chi-squared tests were used to assess associations between categorical variables. No imputation or adjustment was made for missing data. No specific methods were used to address potential sources of bias.

Ethical considerations

Formal ethical approval was not required for this study, as it involved the voluntary and anonymous participation of NHS doctors and did not include any patient data.

## Results

The mean time to complete the questionnaire was three minutes and 12 seconds.

Participant demographics

Fifty-three clinicians completed the questionnaire. Respondents included consultants (n = 6, 11%), specialty registrars (n = 30, 57%), core surgical trainees (n = 14, 26%), GP trainees (n = 1, 2%), and foundation doctors (n = 2, 4%). The majority of respondents (n = 29, 55%) worked in the West Midlands. All other regions of the United Kingdom were represented within the remaining responses (n = 24, 45%).

Main outcomes

Awareness of Guidelines

**Figure 1:**
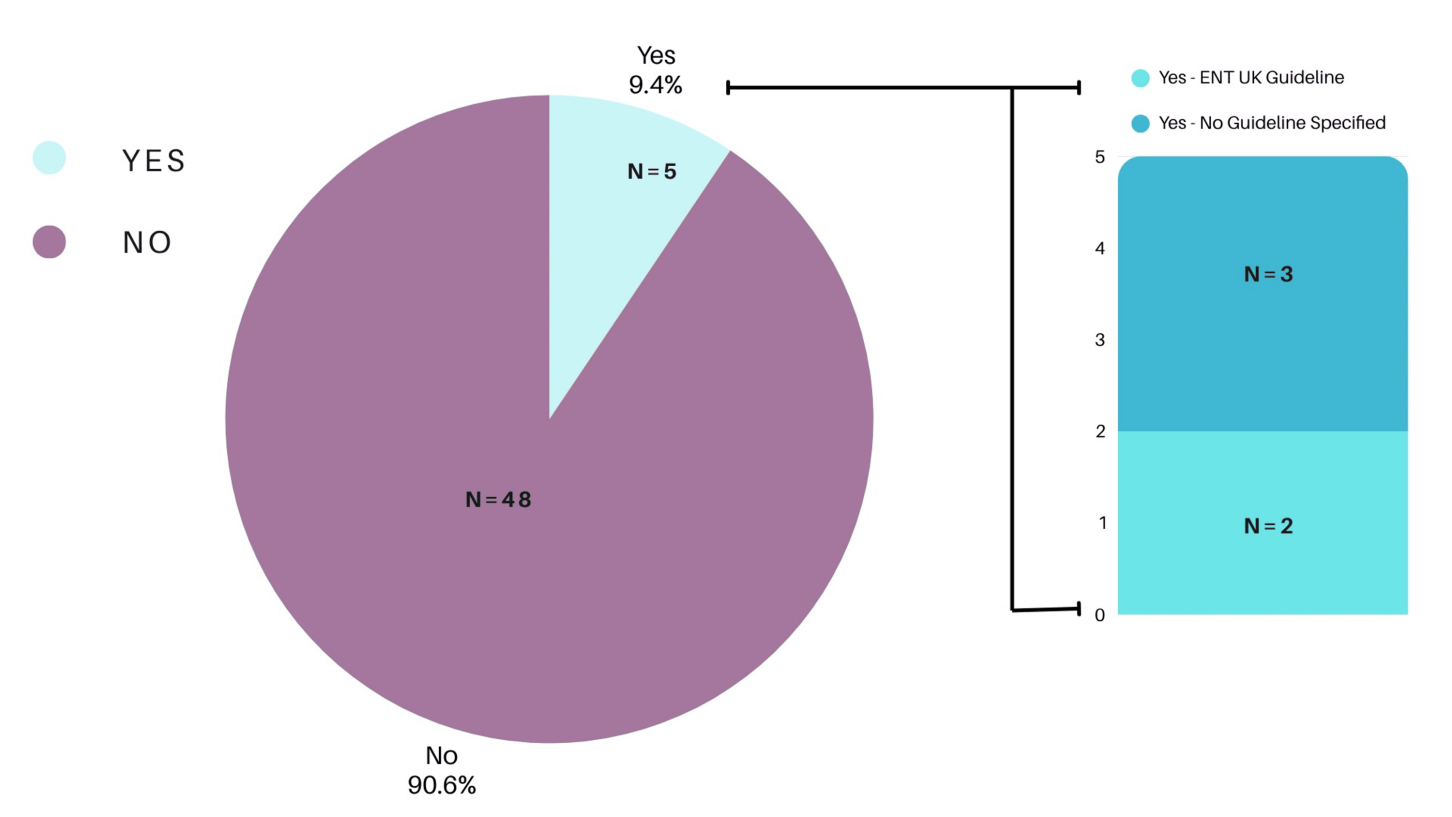
Awareness of ENT UK guidelines. ENT: ear, nose, and throat

Nine percent of respondents (n = 5) reported an awareness of guidelines pertaining to the management of fish bone foreign bodies. Of these, 60% (n = 3) correctly identified the ENT UK guideline (Figure 1).

Confidence in Management

**Figure 2:**
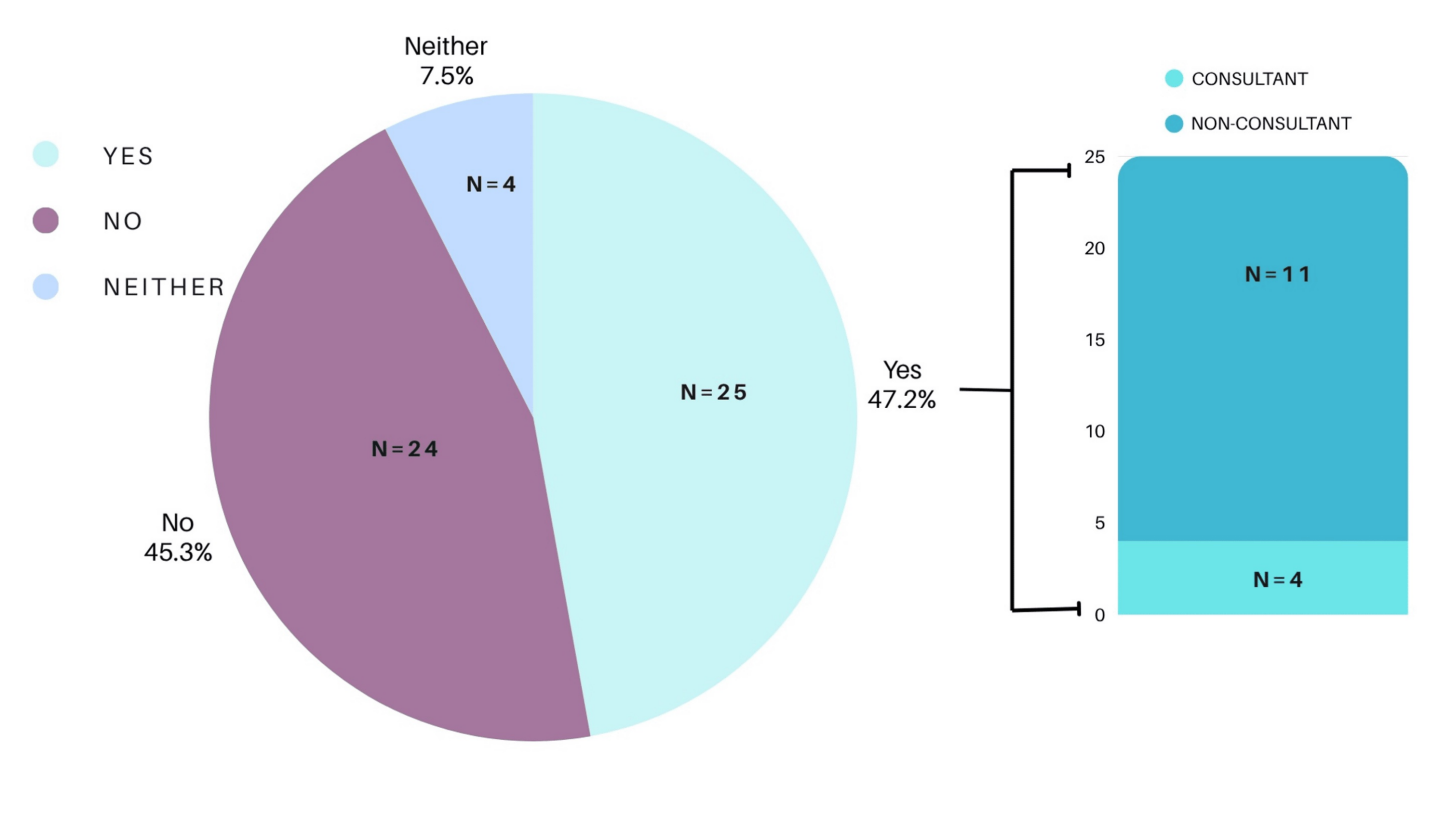
Confidence in the management of fish bone foreign body.

Forty-seven percent of participants (n = 25) reported feeling confident in managing fish bone foreign bodies. Of those who reported feeling confident, 11 out of 25 (44%) were non-consultant grade clinicians (Figure 2). Among consultant respondents, confidence in management was notably higher, with four of the six consultants (66.6%) reporting confidence. However, the association between seniority and confidence was not statistically significant (χ²(1, N = 53) = 1.02, p = 0.31).

Presenting Symptom

**Figure 3:**
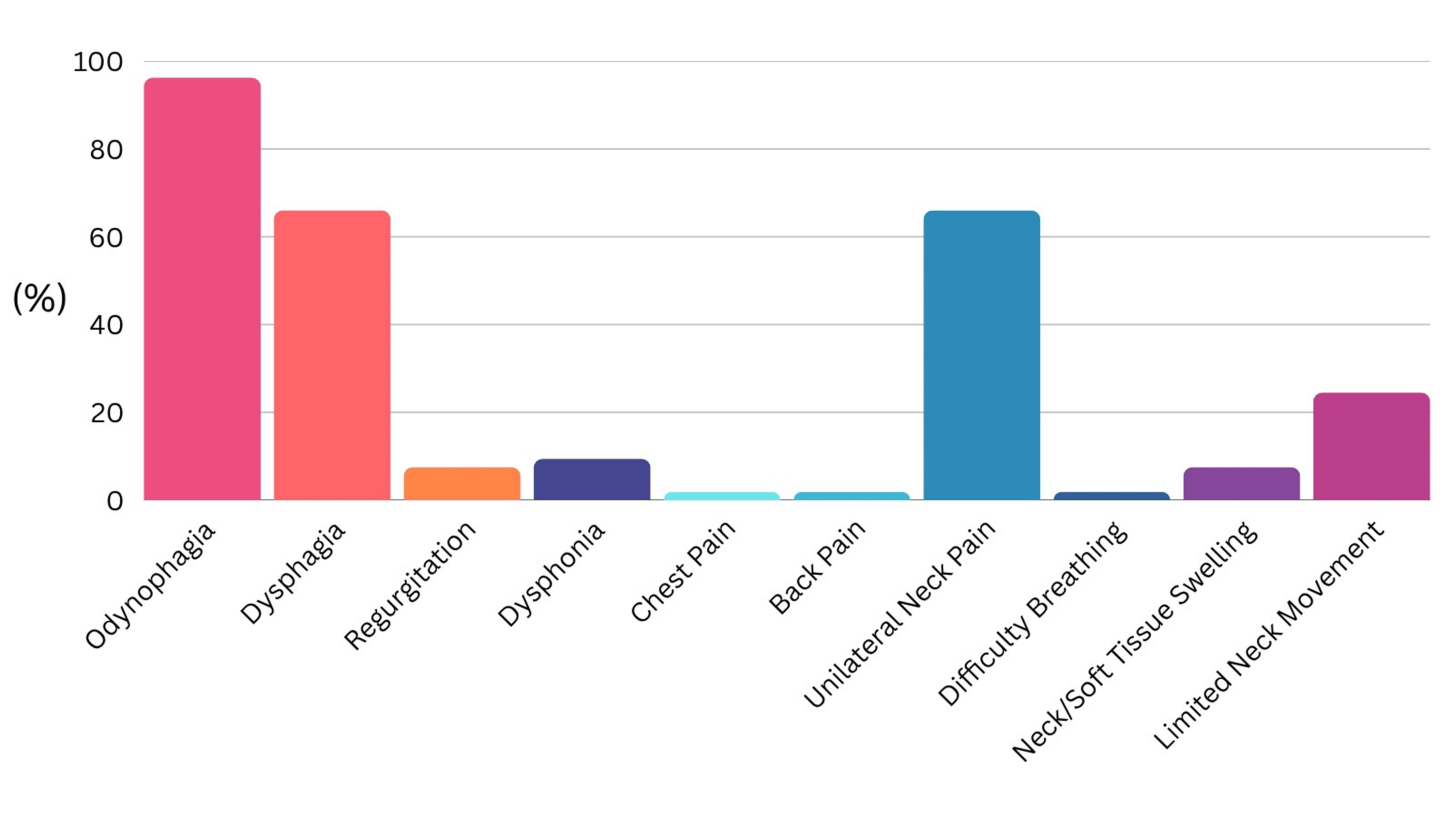
Most commonly encountered presenting symptoms of fish bone foreign body.

The most commonly reported presenting symptoms were odynophagia (n = 51, 96%), dysphagia (n = 35, 66%), and unilateral neck pain (n = 35, 66%). The other reported presenting symptoms are summarized in Figure 3.

Physical Examination Findings

**Figure 4:**
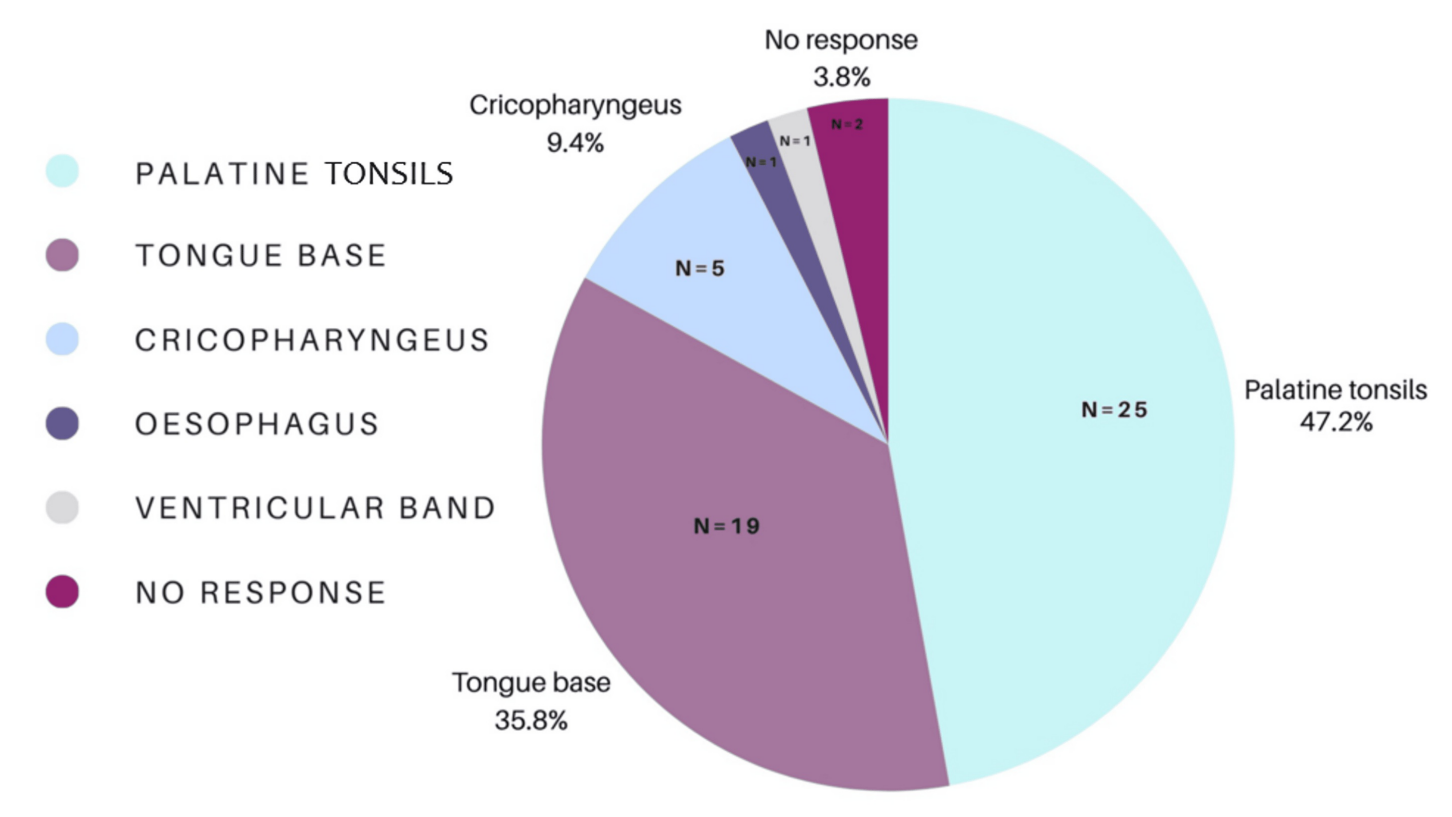
Most commonly encountered locations of fish bone foreign body.

The palatine tonsils (n = 25, 47%) and the tongue base (n = 19, 36%) were reported as the most common sites of fish bone lodgment. Less frequently, respondents reported fish bones lodging in the cricopharyngeus (n = 5, 9%), ventricular band of the larynx (n = 1, 2%), and esophagus (n = 1, 2%) (Figure 4).

Radiography Findings

**Figure 5:**
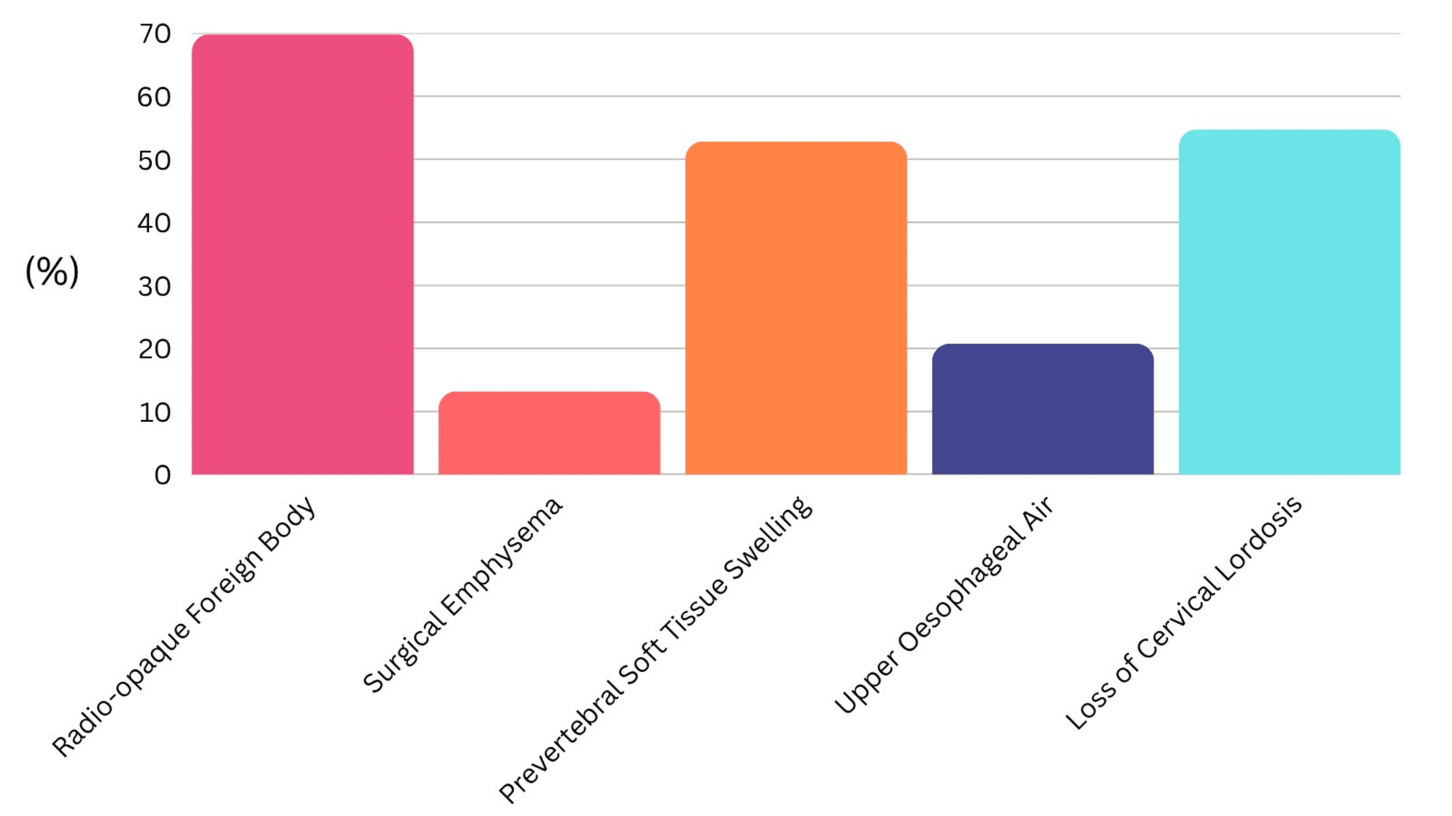
Most commonly encountered positive radiological finding with fish bone foreign body.

Participants could select multiple findings; the most frequently recognized features on lateral soft tissue neck X-rays were a visible foreign body (n = 37, 70%), loss of cervical lordosis (n = 29, 55%), and prevertebral soft tissue swelling (n = 28, 53%). Surgical emphysema (n = 7, 13%) and upper esophageal air (n = 11, 21%) were less commonly identified (Figure 5).

Knowledge of Fish Bone Radio-Opacity

When asked to identify radio-opaque fish species, 66% of respondents (n = 35) correctly identified species such as cod, haddock, and salmon. Conversely, only 11% (n = 6) exclusively identified radiolucent species correctly. Common misconceptions included the classification of trout and mackerel, which were incorrectly identified by a significant proportion of respondents.

Investigative Strategies Following Negative Initial Assessment

When clinical suspicion remained high despite negative findings on FNE or lateral neck X-rays, 66% of respondents (n = 35) selected CT as their next investigation. Other approaches included urgent out-of-hours FNE (n = 7, 13%), non-urgent FNE (n = 5, 9%), discharge with safety-net advice (n = 3, 6%), MRI (n = 1, 2%), and examination under anesthesia (n = 2, 4%). If CT imaging also failed to identify a foreign body but clinical suspicion remained, respondents indicated a preference for examination under anesthesia (n = 18, 34%) or discharge with safety-net advice (n = 12, 23%).

Reported Complications

**Figure 6:**
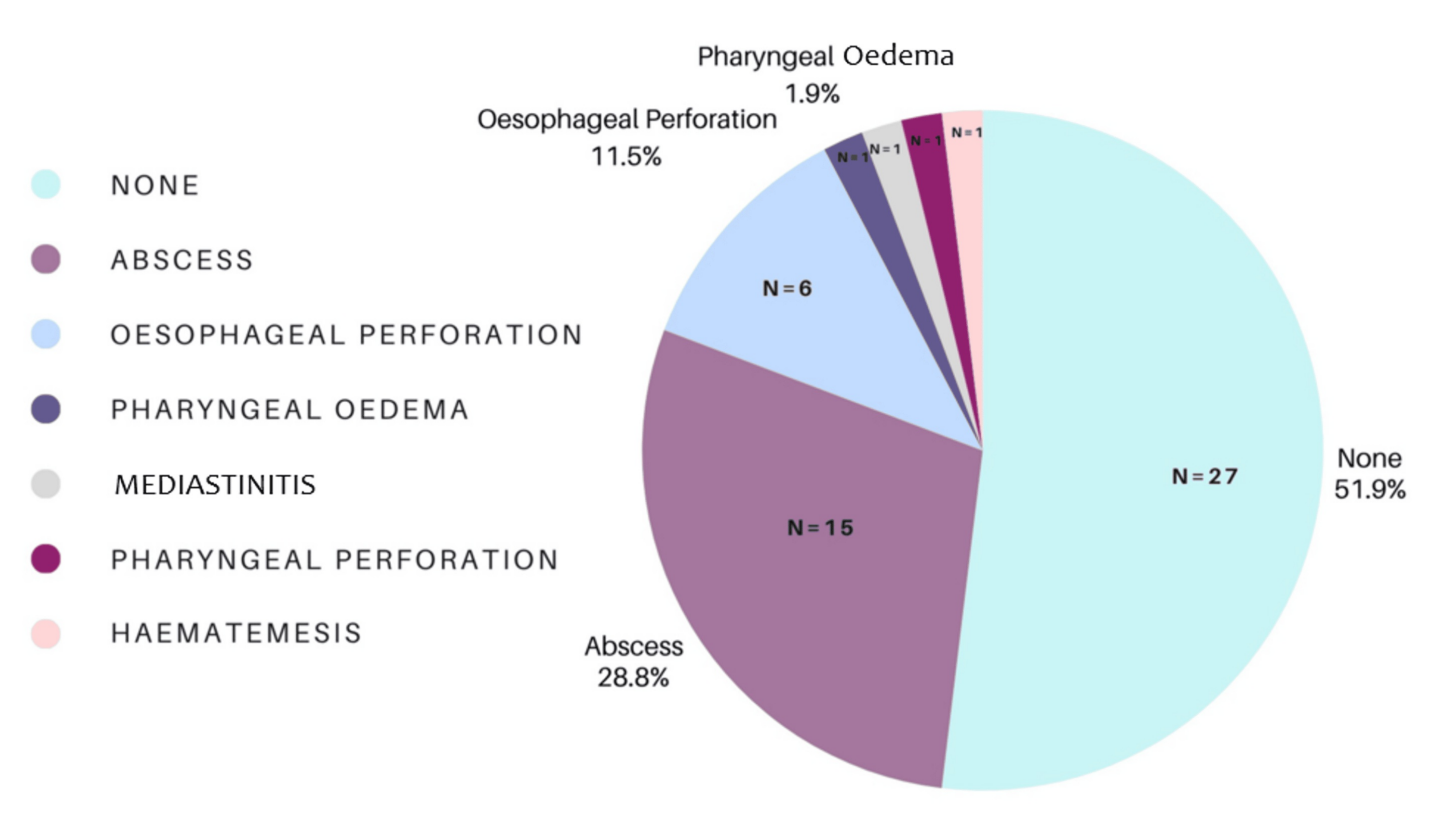
Most commonly encountered complication of fish bone foreign body.

A majority of respondents (n = 27, 51%) had not encountered complications related to retained fish bones. Of those who had (n = 26, 49%), reported complications included abscess formation (n = 15, 28%), esophageal perforation (n = 6, 11%), pharyngeal edema (n = 1, 2%), and hematemesis (n = 1, 2%) (Figure 6).

## Discussion

Several key areas for improvement in the assessment and management of suspected fish bone foreign bodies have been identified. Only 9% of clinicians (n = 5) were aware of relevant guidelines, and 47% (n = 25) reported confidence in managing fish bone foreign bodies.

Awareness of national guidelines was low, consistent with prior reports [16]. Confidence in management was similarly variable, although it was higher among consultants. Interestingly, there was no statistically significant association between seniority and confidence, suggesting that exposure to and education about fish bone foreign bodies may be inconsistent throughout training; however, this finding should be interpreted cautiously given the limited sample size and uneven regional distribution.

Clinical presentation remains a critical diagnostic clue. In this study, odynophagia, dysphagia, and unilateral neck pain were the most frequently reported symptoms, consistent with the existing literature [17]. The palatine tonsils and tongue base were the most common anatomical sites of impaction, again consistent with previous reports [17]. These findings underscore the importance of thorough oropharyngeal examination as a necessary adjunct to FNE [18,19]. Alongside these examinations, the neck should be palpated to exclude surgical emphysema, which is commonly caused by perforation of a viscous and has also been reported secondary to purging [20]. FNE will reveal the most penetrating bones if the endoscope is correctly positioned. It is subject to limitations, specifically operator experience and variable tolerance by patients, which can be mitigated by recording and escalating findings. A previous study found minimal added benefit of FNE in low-risk patients, while highlighting its importance, alongside CT, in medium- and high-risk groups [16].

The use and interpretation of radiological investigations were variable and revealed gaps in knowledge. Although a majority recognized the utility of lateral neck X-rays and CT imaging, understanding of specific secondary radiological signs was limited. Additionally, the identification of radio-opaque versus radiolucent fish species was inconsistent, which may impact decisions regarding the appropriate imaging modalities and highlight the limitations of using this as an investigative tool. It also provides limited assistance in patients presenting with uncertainty about the type of fish they have consumed. Ideally, a radio-opaque fish bone can be directly visualized on X-ray; however, when no bone is visible, secondary radiological features such as prevertebral soft tissue swelling, upper esophageal air, or loss of cervical lordosis may guide management [7,9,21]. Their diagnostic accuracy is variable, with a significant false-negative rate [5,16,17]. A study in New Zealand demonstrated that very few fish species could reliably be identified as radio-opaque on plain film [14]. Similarly, the radio-opacity of commonly cooked British fish species is variable [15]. A retrospective study from Southampton showed that of 60 patients who underwent lateral neck X-ray, 44 scans were interpreted as negative; however, 13 of these patients were later found to have a fish bone on FNE and a further two in the theater [22]. Similarly, a literature review into lateral neck X-rays has shown poor sensitivity. Across seven studies, 57 lateral neck X-rays identified only a single fish bone. The authors suggest that lateral neck X-rays should not be used in routine cases. Instead, they should be reserved for cases in which complications are expected [23]. The low sensitivity of lateral neck X-rays can lead to clinical errors, inappropriate reassurance, and discharge of patients, particularly by junior clinicians.

Our survey revealed that CT was the preferred investigation when the initial assessment was inconclusive. CT imaging has been shown to offer high sensitivity and specificity for detecting ingested fish bones [5]. With one paper suggesting 100% sensitivity, low-dose contrast CT should be considered when the initial assessment is inconclusive but clinical suspicion persists [24]. Low-dose contrast CT has previously been suggested to balance diagnostic benefit and radiation risk, particularly in medium- and high-risk cases [16]. Additional studies have supported the use of low-dose contrast CT over X-rays [25,26]. CT has also identified fish bones in delayed presentations in which panendoscopy was reportedly normal [27].

Panendoscopy can be performed in cases of persistent foreign body sensation without an identifiable fish bone. However, surgery in this circumstance should be pursued judiciously. Rigid esophagoscopy carries a risk of esophageal perforation, particularly when there is no surgical target. Whether the risks of a negative panendoscopy exceed those of a small, retained fish bone is not firmly established.

Complications related to fish bone impaction, although uncommon, were encountered by a substantial proportion of respondents. Abscess formation and esophageal perforation were the most frequently reported complications, aligning with the existing literature [23]. With experience, the clinician’s index of suspicion to detect a genuinely retained fish bone improves. The differential diagnosis is trauma to the pharyngeal mucosa. Both groups are convinced that a fish bone is lodged. Attitudes toward retained fish foreign bodies vary. When foreign body sensation persists without a locatable bone, some clinicians advocate discharge with safety-net advice. The authors' experience is that some ENT colleagues believe small, retained fish bones dissolve over time without intervention. However, this appears to be anecdotal, and we have found no evidence in the literature to support it. Significant morbidity is reported with retained fish bones. A bone in the retropharynx has caused a hematoma threatening the airway, requiring tracheostomy [28]. Retained esophageal fish bones have eroded into the surrounding vascular structures. A fish bone has provably caused rupture of the aortic arch with formation of a pseudoaneurysm, causing fatal hematemesis [29]. Even fish bones that have passed into the stomach can cause complications. Cases of migration into surrounding structures, including the liver, have occurred. Subsequent abscess formation necessitated laparotomy [30]. Given the potential complications associated with missed or retained fish bones, improving knowledge and standardizing management pathways are essential [7], emphasizing the need for vigilance and appropriate escalation strategies, including consideration of examination under anesthesia when imaging and FNE are inconclusive.

Limitations

As a voluntary and anonymous electronic survey, this study is subject to potential selection bias. As the total number of clinicians who received the survey invitation was not recorded, the response rate could not be calculated, and potential response bias cannot be excluded. The use of self-reported data introduces the possibility of recall and social desirability bias, particularly in questions relating to confidence and knowledge. Additionally, the survey employed hypothetical clinical scenarios that may not fully capture the complexity of real-world decision-making. The questionnaire, although detailed, was not pretested or validated, which may affect the reliability of responses. We also acknowledge that a majority of responses originated from the West Midlands region, which may limit national representativeness.

Despite these limitations, the survey provides valuable insights into patterns of practice across multiple training levels. Broader participation and the use of validated instruments in future studies may help to better define national practice and inform targeted educational interventions.

Recommendations

**Figure 7:**
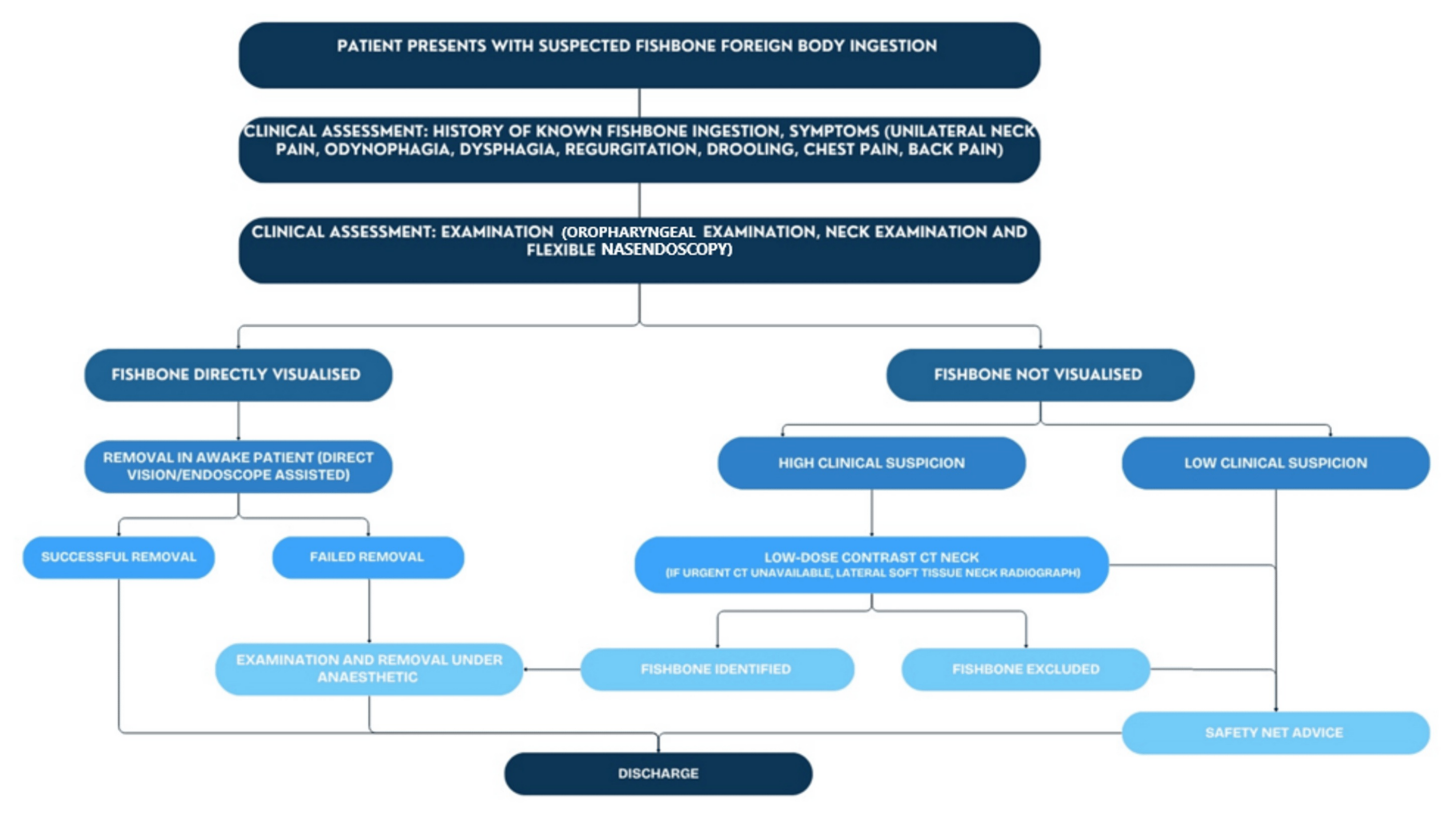
Recommended management of ingested fish bone foreign body.

We propose a revised guideline for the assessment and management of patients presenting with suspected fish bone ingestion (Figure 7).

All patients should undergo an initial history and clinical examination, including oropharyngeal inspection, neck examination, and FNE. If a fish bone is directly visualized, removal in the awake patient under direct vision or with endoscopic assistance should be performed. Successful removal allows for safe discharge, whereas unsuccessful removal requires examination and removal under anesthesia. If no fish bone is visualized, management depends on the degree of clinical suspicion. In cases of low suspicion, patients may be discharged with clear safety-netting advice. If suspicion remains high, a low-dose contrast CT scan of the neck should be performed. When an urgent CT scan is unavailable, a lateral soft tissue neck X-ray can be performed as the initial step. If an X-ray or CT confirms the presence of a foreign body, examination and removal under anesthesia are indicated. Conversely, if CT excludes a foreign body, the patient may be safely discharged with appropriate safety-netting advice.

At each stage of clinical assessment, patients should be counseled on warning signs of complications, such as perforation or mediastinitis, and advised to seek urgent medical attention if these occur, especially where discharge is considered. Amendment to and enhanced education surrounding the guidelines could lead to a reduction in unnecessary investigations, minimize radiation exposure, improve time to treatment, and more accurately diagnose fish bone foreign bodies.

## Conclusions

Fish bone foreign bodies in the upper aerodigestive tract remain a common and potentially serious presentation. There is a significant lack of confidence and consistency among ENT clinicians in the investigation and management of these cases. These findings underscore the urgent need to revise existing post-COVID protocols. We recommend a comprehensive ENT examination, including FNE examination, for all referrals. In cases of persistent diagnostic uncertainty following FNE, low-contrast CT should be favored over lateral neck soft tissue X-rays, which are frequently inconclusive and difficult to interpret. Standardizing these practices through updated guidelines could streamline care, reduce unnecessary investigations, and ultimately improve patient outcomes. Targeted teaching sessions, incorporating guideline summaries into local protocols, and early senior support may enhance clinician confidence, diagnostic accuracy, and patient outcomes. A repeat survey in the future would be valuable to assess the impact of these recommendations and any improvements in clinical practice. As this was a descriptive survey with uneven regional representation, the associations observed should be interpreted cautiously. A repeat national survey with broader participation would be valuable to assess the impact of these recommendations and identify any subsequent improvements in clinical practice.

## Appendices

Fish Bone Foreign Body Management in the United Kingdom: A National Survey

1. What is your level of training?

(Select one)

Foundation year doctor

GP specialty trainee

Core surgical trainee

Higher specialty ENT trainee

ENT consultant

2. In which region do you work currently?

(Select one)

Scotland

North East

North West and Mersey

Yorkshire and Humber

Northern Ireland

Ireland

Wales

West Midlands

East Midlands

East of England

Thames Valley

North Thames

South Thames

South West

Wessex

Kent, Surrey, and Sussex

3. In your clinical experience, what symptoms have confirmed fish bone foreign bodies presented with most commonly?

(Select all that apply)

Odynophagia

Dysphagia

Regurgitation

Change in voice

Chest pain

Back pain

Unilateral neck pain

Difficulty breathing

Neck/soft tissue swelling

Neck stiffness/limited neck movement

4. Which of these fish bones are radio-opaque on X-ray?

(Select all that apply)

Cod

Trout

Salmon

Pike

Haddock

Mackerel

Plaice

Herring

Kipper

Gurnard

Coley

Saithe

5. Which of these fish bones are radiolucent on X-ray?

(Select all that apply)

Cod

Trout

Salmon

Pike

Haddock

Mackerel

Plaice

Herring

Kipper

Gurnard

Coley

Saithe

6. Which of the following radiological signs have you encountered on lateral neck X-ray most frequently in confirmed cases of fish bone?

(Select all that apply)

Opaque foreign body

Surgical emphysema

Soft tissue swelling

Upper esophageal air

Loss of cervical lordosis

Other (please specify)

7. In your practice, where have you most commonly found fish bone foreign bodies?

(Select one)

Tongue base

Soft palate

Palatine tonsil

Ventricular band of larynx

Esophagus

Cricopharyngeus

Other (please specify)

8. If no fish bone foreign body is seen on FNE or lateral neck X-ray, but clinical suspicion is present, what is your next management step?

(Select one)

Non-urgent FNE by a more senior member of the team in-hours/delay until next day if out of hours

Urgent FNE by a more senior member of the team, including out of hours

CT imaging

MRI

Examination under anesthesia (EUA)

Discharge with safety-net advice

9. If no fish bone foreign body is seen on the investigation selected in Question 8, but clinical suspicion is still present, what is your next management step?

(Select one)

Non-urgent FNE by a more senior member of the team in-hours/delay until next day if out of hours

Urgent FNE by a more senior member of the team, including out of hours

CT imaging

MRI

Examination under anesthesia

Discharge with safety-net advice

Not applicable (taken straight for EUA in Q8)

10. Are you aware of any guidelines on fish bone foreign body management?

(Select one)

No

Yes

If yes, please specify: _____________

11. Have you witnessed any complications from fish bone ingestion?

(Select one)

No complications encountered

Yes, esophageal perforation

Yes, localized abscess formation

Yes, hematemesis

Other (please specify)

12. How do you rate your own confidence in managing inhaled fish bones?

(Select one)

I am confident in the management of inhaled fish bones

I am neither confident nor unconfident in the management of inhaled fish bones

I lack confidence in the management of inhaled fish bones
